# Action status and willingness to change health-promoting behaviors during the COVID-19 pandemic among elementary school children: a study based on Prochaska’s stages of behavior change theory (TTM)

**DOI:** 10.1186/s12889-023-15178-w

**Published:** 2023-02-06

**Authors:** Aziz Kamran, Parviz Aliakbari, Ramin Nasimi Doost Azgomi, Mahdi Naeim

**Affiliations:** 1 Social Determinants of Health Research Center, Ardabil University of Medical Sciences, Ardabil, Iran; 2 Traditional Medicine and Hydrotherapy Research Center, Ardabil University of Medical Sciences, Ardabil, Iran

**Keywords:** Lifestyle, Self efficacy, Health promotion, Behavior change, COVID-19

## Abstract

**Background & aim:**

COVID-19 pandemic has significant effects on lifestyle and health-promoting behaviors and adolescence is a very critical period due to the importance of identity formation and individual behaviors. Therefore, the aim of this study was to determine the status of health- promoting behaviors in the sixth grade male students attending elementary schools in Ardabil city based on Prochaska ‘s stages of behavior change (TTM).

**Materials & methods:**

This analytical cross-sectional study was conducted on the 619 sixth grade male students attending elementary schools during 2021. The data were collected using two instruments (a researcher-made questionnaire and Health Promoting Lifestyle Profile-II (HPLP-II) questionnaire). The data were analyzed using SPSS version 22 and one-way analysis of variance.

**Results:**

The majority of students in physical activity and healthy eating behaviors were in the pre-contemplation stage, 29.6% (183 people) and 33.1% (205 people), respectively. All dimensions of health-promoting behaviors were observed to have a significant relationship with the stages of Prochaska’s theory behavior change in students, their decision-making balance and self-efficacy scores (P < 0.001).

**Conclusion:**

The health of students is under serious threat due to the poor state of health-promoting behaviors and the weak desire to change behavior. By planning in educating the students, significant changes can be made in changing their behavior and improving their health.

## Introduction

Coronavirus disease 2019 (COVID-19) is an infectious disease resulting in numerous mild clinical complications or death that was first reported on December 31 2019, in Wuhan, China, and the World Health Organization(WHO), March 11 2020, declared the disease pandemic [1].

Iran is the first country in the Middle East that reported the outbreak of COVID-19, and also had the highest prevalence of COVID-19 in the region [2] According to the evidence, during the implementation of this study (2021), the prevalence of COVID-19in Iran was 8% and 59,483 people died due to it [3].

Around the world, several government policies have been implemented to control the COVID-19, including social distancing and travel restrictions, closing schools and sports clubs, which can have a large impact on public health-promoting behaviors, including children and adolescents [4–6].

Adolescence is an important and vital period that is associated with physical, emotional and developmental changes and prepares a person to enter adulthood, and also the combination of these changes and their effect on diet, sleep, exercise and physical activity (PA), weight control, etc., shapes the health-related behaviors in adolescents and ultimately affects lifestyle [5] Lifestyle is Health-related behaviors can results from lifestyle, and also they include a wide range of actions that simultaneously or in the future can have a positive or negative impact on a person’s health status [7, 8].

Health-promoting behaviors (HPBs) have important health implications, and the risks associated with participating in any particular behavior may increase or decrease depending on other behaviors that the individual engages in and groups that include several unhealthy behaviors are more common among adolescents than older age groups [9].

A similar study performed in Iran showed that PA is more unfavorable than other dimensions [4]. In another study conducted in Iran on teenagers, PA and stress management had the lowest scores [10].

Ardabil province is located in the northwest of Iran and has a population of about 1.3 million [11].

Recently, a similar study conducted on 30 provinces of Iran showed high prevalence of insufficient PA in the entire population (54.7%). In addition, insufficient consumption of fruits and vegetables was associated with insufficient PA [12]. Also, other studies performed in Iran showed that the prevalence of hypertension varies between 25 and 35% and obesity, waist size, inactivity, high stress are the main causes of high blood pressure [13]–[14].

Despite the adoption of pandemic control policies, including complete and partial quarantine, the evidence shows the serious effects of the pandemic on mental health, quality of life and depression in students [15]. Previous studies have shown that the high prevalence of anxiety (34–37%), depression (43–45%) and combined symptoms of anxiety and depression (31%) in students during the Covid-19 epidemic [16]–[17]. And other similar studies showed that COVID-19 has negative effects on mental health and leads to anxiety and depression [18]–[19].

In a situation where the world is facing the COVID-19 pandemic, the synergy of other risk factors for chronic diseases, such as obesity, malnutrition, inactivity, poor mental health and health literacy can provide more serious threats to the human health. This synergism of the epidemic and the risk factors for chronic diseases requires the development of children’s health literacy and the empowerment of Anna to promote a sense of responsibility [20].

The emergence of non-communicable diseases (NCD) as a new challenge for Iran’s health care doubles the need to pay attention to HPBs and recent evidence shows that in 2019, 15.5 million DALYs were attributed to NCD, which is 44.2% higher than in 1990. Ischemic heart disease was the leading cause of age-standardized DALY rates in 2019, and the next leading causes were stroke, diabetes, back pain, and depressive disorders at the national level and in most provinces in 2019 [21] .

Numerous theories can explain the involvement of the individual in adopting a new behavior, the Trans theoretical model (TTM) is an integrated and comprehensive model of behavior change that has been widely used to promote exercise and nutrition behaviors [22], according to this model, behavior change is not an event but a process and people are involved in different stages of the change process. These stages of change include pre-contemplation, contemplation, preparation, action, and maintenance. Using behavior change steps to understand the mechanism of exercise behavior and nutrition can determine an individual’s readiness to change and determine the relationship between different structures which can predict the probability of adoption and success in adopting and maintaining exercise behaviors [23].

To the best of our knowledge, few studies have been conducted on HPBs among adolescents after the COVID-19 pandemic, and this is the first study in Iran to examine PA and healthy eating in Iranian children. Given the importance of the subject; therefore, the aim of this study was to determine the status of HPBs of the sixth grade male students attending elementary schools in Ardabil and its association with the Prochaska ‘s stages of behavior change.

## Materials & methods

This analytical cross-sectional study was conducted on 619 the sixth grade male students attending elementary schools in Ardabil city who met the study inclusion criteria and were randomly selected.

The study questionnaire is a tool consisting of 6 sections as follows. To collect the data, first, the objectives of the study were explained to the participants, school principals and parents of students. Then, the questionnaire was completed as a face-to-face interview. To maintain the confidentiality of the information, the questionnaires were anonymous. The inclusion criteria for this study were as follows: not suffering from limb disability and movement limiting disease, no surgery in the last 6 months, no treatment under the supervision of a psychologist and psychiatrist, studying in the 6th grade, and the age of 12 and 13. Also, people who were unwilling to complete the interview were excluded from the study. The data was collected from March to September 2021.

The first part of the questionnaire included personal information questions (such as field of study, number of children in the family, birth rank, father’s education level, mother’s education level, father’s job, mother’s job, father’s age, and mother’s age). The second part of the tool was a researcher-made questionnaire using the structures of stages of behavior change, including two questions to determine the stage of change in nutritional behaviors and physical activity, indicating that each of the participants stated the status of their decision or action regarding compliance healthy diet and physical activity (pre-contemplation: do not intend to take action within the next 6 months, contemplation: intending to start the healthy behavior within the next 6 months, preparation: ready to take action within the next 30 days, Action: have recently changed their behavior within the last 6 months, and maintenance: sustained their behavior change more than 6 months). The third part of questionnaire was the Health-Promoting Lifestyle Profile (HPLP-II) questionnaire. The fourth part of the questionnaire consisted of 12 questions about the balance of decision making. The fifth section of the tool included 11 questions about students’ self-efficacy to assess their ability to take action for HPBs, with scores ranging from 1 (not important at all) to 5 (very important).

The face validity of the researcher-made tool was provided based on the opinions of health and nursing education experts. Content validity was also assessed based on CVI and CVR indices. Its reliability was 0.9 using Cronbach’s alpha after confirming its validity.

The HPLP II questionnaire consists of the 52 items of health-promoting behaviors with 6 dimensions, including responsibility (n = 12 items), physical activity (measuring regular patterns of exercise and Physical exercise, n = 7 items), nutrition (evaluation of dietary patterns and nutritional choices, n = 9 items), spiritual growth (evaluation of spiritual growth, n = 11items), stress management (measurement of coping ability, n = 8 items) and interpersonal relationships (determining the feeling of intimacy and relationship, n = 5 items). Each item is scored on a 4-choice Likert scale, including never (1 point), sometimes (2 points), usually (3 points), and always (4 points). The total score range of HPBs is a minimum of 52 and a maximum of 208, and for each dimension, a separate score can be calculated, and a higher score indicates better HPBs. The reliability of this tool in Persian studies has been reported to be higher than 0.8 for the whole tool and isdimensions [24].

## Ethical considerations

The license of the ethics committee of Ardabil University of Medical Sciences was obtained for the mentioned dissertation with the number IR-ARUMS-REC 1398.78.

## Method of data analysis and statistical analysis

Data were analyzed by descriptive and analytical statistics using SPSS version 22 and ANOVA and chi-square test for quantitative data. The significance level was considered less than 0.05.

## Results

The results of this study showed that the majority of parents had secondary education level, most father were employee of governmental organizations and other occupations and the majority of mothers were housewives (Table 1).

**Table 1:** Frequency distribution of parents’ education level in the students

	Job	F	%
**Father**	illiterate	43	6.9
	Middle school	123	19.9
	High school	200	32.3
	Bachelor	152	24.6
	Higher than a bachelor’s degree	101	16.3
	Total	619	100
**Mother**	illiterate	47	7.6
	Middle school	122	19.7
	High school	240	38.8
	Bachelor	161	26
	Higher than a bachelor’s degree	49	7.9
	Total	619	100
**Father**	Employee	195	31.5
	Shopkeeper	79	12.8
	Farmer / rancher	27	4.4
	Driver	53	8.6
	Unemployed	21	3.4
	Other	244	39.4
	Total	619	100
**Mother**	Employee	75	12.1
	Shopkeeper	12	1.9
	housewife	502	81.1
	Freelance	30	4.8
	Total	619	100

**Table 2:** Distribution of students’ frequency in the decision stages to plan exercise and healthy nutrition

	Items	F	%
**Exercise**	I have not made a decision yet and it is not in my plan	183	29.6
	I plan to start exercising in the next 6 months	96	15.5
	I plan to start exercising next month	103	16.6
	I have been exercising regularly for the last 6 months	88	14.2
	I have been exercising regularly for more than 6 months	149	24.1
**Nutrition**	I have not made a decision yet and it is not in my plan	205	33.1
	I plan to start feeding in the next 6 months	50	8.1
	I plan to start feeding next month	83	13.4
	For the past 6 months, I have started to eat regularly	88	14.2
	I have been eating regularly for more than 6 months	193	31.2
Total		619	100

The majority of students in PA (n = 183, 29.6%) and healthy eating (n = 205, 33.1%) were in the pre-contemplation stage of the Projaska’s behavior change model. (Table 2)

**Table 3:** The mean and standard deviation of health promoting behaviors dimensions in the students. The mean scores of the responsibility and spiritual growth in students were higher than those of other dimensions. (Table 3)

Variable	Mean	SD	Maximum achievable score	Maximum	Minimum
Balance of decision	29.27	8.02	44	44	11
Self Efficacy	19.20	4.95	24	24	6
Spiritual growth	33.38	7.35	40	40	10
Responsibility	35.25	11.35	48	48	12
Interpersonal relationships	24.65	7.04	32	32	8
Stress	14.26	4.89	20	20	5
Exercise and physical activity	16.91	5.99	24	24	6
Nutrition	21.11	6.07	28	28	7

**Table 4:** Mean scores of decision balance, self-efficacy, spiritual growth, and responsibility by stages of changing the behavior of exercise activity

		Balance of decision	Self Efficacy	Spiritual	Responsibility
		Mean ± SD	Mean ± SD	Mean ± SD	Mean ± SD
**Exercise**	I have not made a decision yet	19.8 ± 3.52	12.8 ± 3.52	23.8 ± 5.41	20.7 ± 5.34
	Start in the next 6 months	25.2 ± 1.82	18.3 ± 0.53	32.5 ± 2.32	31.3 ± 3.19
	Starting next month	31.07 ± 1.92	20.4 ± 1.21	36.8 ± 0.38	38.4 ± 1.17
	I started from the last six months	34.0 ± 0.23	23.6 ± 0.46	39.0 ± 1.39	44.3 ± 2.96
	More than 6 months	39.3 ± 3.21	24.0 ± 0.00	40.0 ± 0.00	48.0 ± 0.00
	Total	29.27 ± 8.02	19.2 ± 4.95	33.3 ± 7.35	35.2 ± 11.35
P - value		P < 0.001	P < 0.001	P < 0.001	P < 0.001

As shown in Table 4, over 6 months, the results showed that people who were in the upper stages of Prochaska’s behavior change reported higher mean scores than lower levels. This means that a person who has been exercising regularly for more than 6 months had significantly higher scores in decision balance, self-efficacy, spiritual growth, and responsibility (p < 0.05).

**Table 5:** Mean scores of interpersonal relationships, stress, physical activity, and nutrition by stages of behavior change in exercise activity

Variable		Interpersonal relationships	Stress	Physical activity	Nutrition
		Mean ± SD	Mean ± SD	Mean ± SD	Mean ± SD
**Exercise**	I have not made a decision yet	15.5 ± 3.94	8.23 ± 2.55	9.4 ± 2.61	13.2 ± 3.28
	Start in the next 6 months	22.59 ± 3.49	12.1 ± 1.11	14.2 ± 1.05	19.45 ± 0.67
	Starting next month	26.6 ± 1.31	15.6 ± 0.62	18.0 ± 1.07	23.04 ± 1.45
	I started from the last six months	31.06 ± 0.42	17.8 ± 1.38	22.03 ± 0.74	25.54 ± 0.78
	More than 6 months	32.0 ± 0.00	20.0 ± 0.00	24.0 ± 0.00	27.88 ± 0.31
	Total	24.65 ± 7.04	14.2 ± 4.89	16.9 ± 5.99	21.1 ± 6.07
P – value		P < 0.001	P < 0.001	P < 0.001	P < 0.001

As shown in Table 5, the results showed that people who were in the upper stages of Prochaska behavior change reported higher mean scores than lower levels. That is, individuals who exercised regularly for more than 6 months had significantly higher scores in interpersonal relationships, stress, physical activity, and nutrition (p < 0.05).

**Table 6:** Mean scores of decision balance, self-efficacy, spiritual growth, and responsibility) by stages of nutritional behavior change

Variable		Balance of decision	Self Efficacy	Spiritual	Responsibility
		Mean ± SD	Mean ± SD	Mean ± SD	Mean ± SD
**Nutrition**	I have not made a decision yet	20.17 ± 3.46	13.4 ± 3.68	24.4 ± 5.46	21.4 ± 5.45
	Start in the next 6 months	25.2 ± 1.37	18.3 ± 0.47	32.2 ± 1.80	31.2 ± 2.16
	Starting next month	28.9 ± 1.24	19.4 ± 0.73	36.3 ± 0.73	36.9 ± 1.31
	I started from the last six months	33.5 ± 0.85	22.4 ± 1.01	37.5 ± 1.09	40.6 ± 1.78
	More than 6 months	38.1 ± 3.58	24.0 ± 0.00	40.0 ± 0.00	47.7 ± 0.62
	Total	29.2 ± 8.02	19.2 ± 4.95	33.3 ± 7.35	35.2 ± 11.35
P – value		P < 0.001	P < 0.001	P < 0.001	P < 0.001

As shown in Table 6, the results showed that people who were in the upper stages of Prochaska behavior change reported higher mean scores than lower levels. This means that individuals who exercised regularly for more than 6 months had significantly higher scores in decision balance, self-efficacy, spiritual growth, and responsibility (p < 0.05).

**Table 7:** Mean score of dimensions of interpersonal relationships, stress, physical activity, and nutrition by stages of changing eating behavior

Variable		Interpersonal relationships	Stress	Physical activity	Nutrition
		Mean ± SD	Mean ± SD	Mean ± SD	Mean ± SD
**Nutrition**	I have not made a decision yet	16.2 ± 4.2	8.5 ± 2.5	9.8 ± 2.7	13.8 ± 3.56
	Start in the next 6 months	22.6 ± 0.47	12.0 ± 0.99	14.1 ± 0.74	19.2 ± 0.45
	Starting next month	24.9 ± 1.34	14.7 ± 1.21	16.8 ± 1.14	21.6 ± 1.35
	I started from the last six months	29.3 ± 1.66	16.3 ± 0.74	20.1 ± 1.49	24.6 ± 0.48
	More than 6 months	31.8 ± 0.37	19.7 ± 0.57	23.6 ± 0.68	27.4 ± 0.89
	Total	24.6 ± 7.04	14.26 ± 4.89	16.9 ± 5.99	21.11 ± 6.07
P – value		P < 0.001	P < 0.001	P < 0.001	P < 0.001

According to the results (Table 7), participants who were in the upper stages of Prochaska behavior change reported higher mean scores than others. This means that a person who has been exercising regularly for more than 6 months has significantly higher scores in decision balance, self-efficacy, spiritual growth, and responsibility (p < 0.05).

## Discussion and conclusion

The results of this study showed that the majority of students are in the first stage of behavior change and have not yet made a decision to improve their HPBs, including PA and healthy eating. While these behaviors are the main causes of NCD, especially cardiovascular diseases. In line with this finding, as the final result of inactivity and unhealthy eating, a recent study published in Iran shows that the total number of DALYs in 2019 reached 19.8 million, and in 2019, 78.1% of disability-adjusted life years (DALYs) were due to NCD, which compared to 1990 had increased by 44% [21].

Other studies in Iran have shown a high prevalence of modifiable cardiovascular risk factors, including obesity and overweight (60%) [25], dyslipidemia (80%) [26], high blood pressure (53%) [27] and it seems that arrive the current policies of primary, secondary and tertiary prevention have not been successful to deal with the challenge of NCD in Iran, and health policy makers should adopt multi-sectoral approaches to reduce risk factors and follow up on the tsunami of deaths due to NCD. The best time and place to implement prevention policies is in elementary schools, where health-related behaviors and personalities are being formed.

In the present study, the total mean scores of the health promotion dimensions (145.5 ± 42.3) and the highest and the lowest scores were related to health responsibility (35.25 ± 11.35) and stress management (14.26 ± 4.89), respectively. In the study of Sami Al-Zahraei (2018), the mean total score (123.8 ± 19.8), the lowest mean score was related to health responsibility [28]. The studies conducted by Moeini (2015) [29] was consistent with our study, but in a study by Shaheen et al. (2014), the highest and the lowest mean scores were related to spiritual growth and PA, respectively [30].

The COVID-19 pandemic had a great impact on the mental health of parents due to job restrictions and reduced income, which can significantly affect parent-child relationships and increase the risk of children’s mental health problems [31].

Although closing schools was the most effective policy to control the COVID-19 pandemic, it had adverse effects on children and adolescents due to the deprivation from education and school attendance [31]. Also, they were exposed to poverty caused by the pandemic and domestic violence caused by the disability of the parents’ in control of mental challenges. These issues were one of the plausible reasons for the findings of the present study that the lowest average score of health promoting behaviors was related to stress management.

Our results showed that in regular PA, the majority of participants were in the pre-contemplation stage (29.6%) and did not have a plan for regular PA in the next 6 months. In pre-pandemic studies of COVID-19, in a Balali ‘s study (2015) in the field of PA (exercise), the highest percentage was related to the operation phase and also in the field of healthy nutrition, the highest percentage was related to the pre-contemplation phase [31]. Also, in the Saadati’s study (2012) in the field of PA, the highest percentage was related to the preparation stage [32]. Moreover, in Lacey’s (2017) study in the field of PA (exercise), the highest percentage was related to the pre-contemplation stage and in the field of nutrition planning, the highest percentage was related to the pre-contemplation stage [33]. The COVID-19 pandemic appears to have reduced PA in students, which is consistent with previous studies conducted by Xiang et al. [6]; Zenic et al. [34]; Guerrero et al. [35]; Lopez Bueno et al. [36]; Elnaggar et al. [37].

Discrepancies in the results may be due to various underlying factors, such as prevention and control strategies of COVID-19 and its prevalence during the study period. Some countries like China, completely banned traffic and imposed severe restrictions, while others, including Belgium, imposed only light restrictions. On the other hand, the differences may be due to problems with the method of data collection in multiple studies.

Also, in terms of healthy eating, the majority of people (n = 205, 33.1%) were in the pre-contemplation stage and did not have a plan to adjust their diet plan for the next 6 months. There is very little evidence of the effect of COVID-19 on adolescent nutrition, but in a Spanish study, the results showed that there was a significant reduction in adolescent fruit and vegetable consumption [36], and also in the study, the consumption of sweet drinks, red meat, and potato chips was increased [38].

Restrictions related to social distance led to the closure of cooking and distribution centers of prepared foods, which could be reduced by decreasing fast-food consumption, such as reducing sugar and excess fat and fiber consumption, [39] as well as by closing schools through deprivation. Healthy eating instructions in school can be effective in changing healthy eating habits [40]. In addition, nutritional behavior is affected by the knowledge, self-regulation, and socioeconomic status of individuals [41].

The effects of the COVID-19 pandemic on income caused a change in food patterns in families, and in addition, it changed the behavior of households such as providing food necessities and even taking loans and buying non-food items [42]. This factor can have serious effects on the stages of changing the nutritional behavior of children in the present study, and children are affected by the behaviors and behavioral patterns of the family.

The results of this study showed that people who were in the maintenance and preparation stages had higher mean scores in HPBs than lower levels. Consistent with the results of the present study, Geok (2015) showed that by increasing the mean scores of all aspects of health promotion, PA was high [43]. Other similar studies have shown that nutritional behavior improves after educational interventions and by improving the stages of behavior change [44].

## Conclusion

Most of the subjects were in the pre-contemplation stage for regular PA and following a healthy diet, which shows that the students did not have a plan to adherence a healthy diet and increase PA in the next 6 months. Students’ intention to follow healthy behaviors had a significant correlation with dimensions of HPBs, and the mean score of people who were in the higher stages of Prochaska’s behavior change theory was significantly higher than other people in the lower stages.

This finding means that paying attention to the concept of intention to change behavior and factors affecting it can be effective in improving HPBs in epidemic conditions. Especially at the age of children in elementary schools, paying attention to this concept can be very effective in the formation of healthy habits in children and reduce the burden of NCD and their mortality. This is while Iran was among the countries that implemented social distancing and partial quarantine in the management of the COVID-19 pandemic, and in the process of providing health care, a special program for children in primary ages was not implemented. Paying attention to the consequences that threaten mental health, including anxiety and stress, changing children’s HPBs, including healthy eating and PA, had been neglected in the health care programs, and the main emphasis was on vaccination, wearing masks, and social distancing.

## Implications

Intention to change behavior is a key factor in adherence to HPBs and the pandemic situation can affect the influencing factors on the intention to change the behavior. The emergence of COVID-19 and the consequences of the pandemic, such as restrictions and social distancing, and the intersection of the consequences of the pandemic with socio-economic gaps, poor health literacy and health responsibility, can have a profound effect on HPBs, which in the current study, the majority of people were in the initial stages of behavior change and had not made a decision to move towards regular exercise and adherence to a healthy diet. These effects can make the crisis of NCD in Iran more serious and have direct and indirect effects on the care provided for major diseases.

Therefore, there should be a need for future studies to evaluate the effects of the pandemic regarding HPBs and dimensions of health perceptions, including self-efficacy and decision-making balance, in order to adopt detailed evidence-based policies to prevent more adverse effects of COVID-19.

## Recommendations

Due to the low mean scores of dimensions of HPBs, including stress management, nutrition and PA, the assessment of physical and mental health status, knowledge of education, mental stress, depression of parents, children’s sleep problems, exposure to media and other related problems are vital to prevent more complications to the mind and body of children and adolescents.

Regular study and monitoring of children’s mental and physical health status, especially in children living in deprived areas, are also suggested to manage emotional, mental and physical crises caused by Covid-19.

## Study limitations

This study, like other cross-sectional studies, has limitations, such as the fact that this study cross-sectionally examines the status of students in HPBs and the stages of changing these behaviors, and it is not possible to answer the role of the pandemic in the results obtained. Therefore, it is suggested that future studies should pay attention to comparing health promoting behaviors before and after the pandemic. Also, this study was only conducted on boys, and considering the greater effects of the pandemic on the mental health of girls, it is recommended to conduct a comparative study between men and women in future studies. Other limitations of this study are due to the prevailing conditions in the society due to the spread of the pandemic and since the questionnaires were completed virtually by the students, there was a possibility of parental guidance and intervention, which is better to pay attention to in the interpretation of the results.

## Data Availability

The data analyzed in the study are available from the corresponding author on reasonable request.
